# A labeled data set of underwater images of fish and crab species from five mesohabitats in Puget Sound WA USA

**DOI:** 10.1038/s41597-023-02557-6

**Published:** 2023-11-13

**Authors:** Dara M. Farrell, Bridget Ferriss, Beth Sanderson, Karl Veggerby, Lauren Robinson, Anusua Trivedi, Shreyaan Pathak, Sreya Muppalla, Jane Wang, Dan Morris, Rahul Dodhia

**Affiliations:** 1 University of Washington (UW), School of Aquatic and Fishery Sciences, Seattle, 98195 USA; 2 Alaska Fisheries Science Center, National Marine Fisheries Service, National Oceanic and Atmospheric Administration (NOAA), 7600 Sand Point Way Northeast, Seattle, 98115 USA; 3 Northwest Fisheries Science Center, National Marine Fisheries Service, National Oceanic and Atmospheric Administration, 2725 Montlake Boulevard East, Seattle, 98112 USA; 4 iMerit, 985 University Avenue, Suite 8, Los Gatos, California 95032 USA; 5 Microsoft (MS), Microsoft AI for Good Research Lab, 14820 NE 36th Street, Redmond, Redmond, 98052 Washington USA

**Keywords:** Behavioural ecology, Environmental impact

## Abstract

The sustainable management of fisheries and aquaculture requires an understanding of how these activities interact with natural fish populations. GoPro cameras were used to collect an underwater video data set on and around shellfish aquaculture farms in an estuary in the NE Pacific from June to August 2017 and June to August 2018 to better understand habitat use by the local fish and crab communities. Images extracted from these videos were labeled to produce a data set that is suitable for use in training computer vision models. The labeled data set contains 77,739 images sampled from the collected video; 67,990 objects (fishes and crustaceans) have been annotated in 30,384 images (the remainder have been annotated as “empty”). The metadata of the data set also indicates whether a physical magenta filter was used during video collection to counteract reduced visibility. These data have the potential to help researchers address system-level and in-depth regional shellfish aquaculture questions related to ecosystem services and shellfish aquaculture interactions.

## Background & Summary

Shellfish are central to Washington State’s culture, marine ecosystems, and coastal economies. Washington is the nation’s leading producer of farmed clams, oysters, and mussels, contributing approximately $229 million to the State economy^1^, and supplying fresh shellfish to consumers around the world. With such high cultural, economic, and ecological value, there is substantial demand for growth within the shellfish aquaculture industry. Limited understanding of the ecological implications of converting nearshore habitat to shellfish production is a key impediment to the sustainable expansion of shellfish aquaculture. An improved understanding of how shellfish aquaculture functions as a nearshore habitat (relative to uncultivated areas) will help resource managers overcome this barrier. Consequently, they will be able to better assess potential trade-offs when planning the sustainable expansion of shellfish aquaculture.

Quantifying habitat use by fishes can be logistically challenging and costly because aquaculture sites often have structures in the water impeding sampling nets, the sampling must occur around high tide when the sites are submerged, and large data sets are required to account for mobility and variability among the nearshore fish community. Underwater video is a promising method of data collection, but its efficacy is limited by the time required to process the video. The benefits of using underwater video include the ability to: sample in complex habitats (i.e., aquaculture farms with structures in the water), collect passive observations (i.e., removing the bias of sampling disturbance found in beach seines and snorkel/dive surveys), and improve the quality of data (e.g., by verifying species identifications versus snorkel/dive surveys in which there is only one chance for identification and counting).

**Fig. 1:**
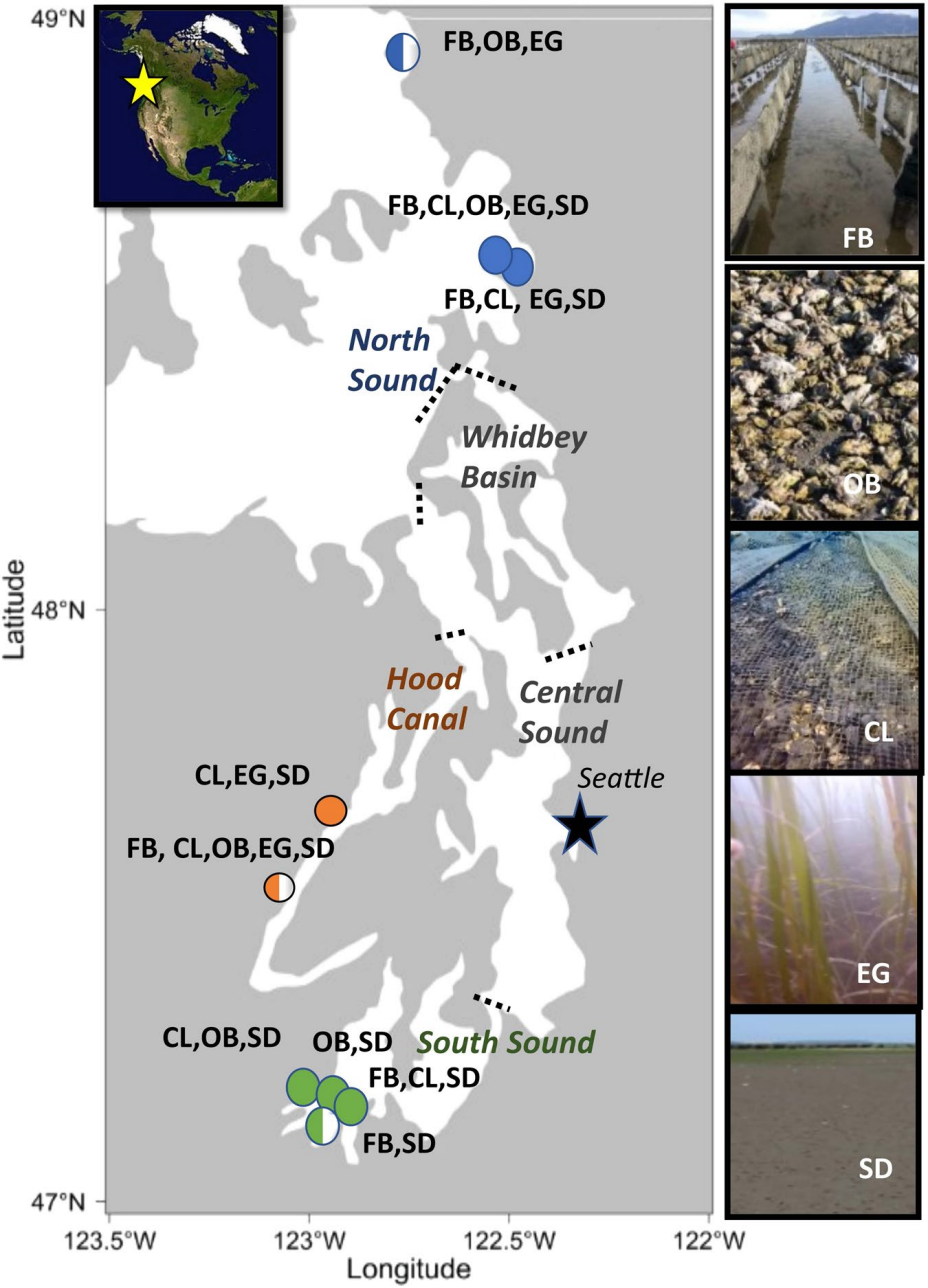
The 9 sample sites in North Sound (blue circles), South Sound (green circles), and Hood Canal (orange circles) in Puget Sound, Washington, USA. The 5 natural basins are labeled with their boundaries marked by dashed black lines. The photos in the figure show the 5 mesohabitat types that were sampled approximately twice per summer in 2017 and 2018: Pacific oysters in flipbags (FB), Pacific oysters on bottom (OB), Manila clams (CL), uncultured eelgrass (EG), and uncultured. In the figure, full circles indicate that a given site was sampled in both years, and half-filled circles indicate that the given site was only sampled in 2018. This figure has been adapted from a previously published figure in Ferriss *et al*.^2^.

This motivated the collection of video data in Puget Sound, a large fjord-like estuary in the northwest USA that is 161 km long with an average depth of 70 m and a 2,143 km coastline. Stratification separates the deep and shallow waters of this estuary because shallow waters tend to be warmer and less dense than deep water, resulting in horizontal layers in the water column (MacCready P., Encyclopedia of Puget Sound, 2017). Puget Sound is comprised of five ecologically distinct sub-basins characterized primarily by geomorphology, extent of freshwater influence, oceanographic conditions, and anthropogenic stressors. Inter-basin differences have been observed for intertidal, subtidal, and nearshore pelagic species in Puget Sound, but no conclusive mechanisms or defining drivers have been identified^2,3^. Video was collected in this estuary in collaboration with shellfish growers from June to August in 2017 and from June to August in 2018 (the summers of 2017 and 2018, respectively) from the locations shown in the main panel of Fig. 1. The video collection study focused on the intertidal zone (the shallow water region bounded by high tide and low tide levels), and was designed based on existing knowledge that the natural distribution of species and habitat types (including mudflat extent and eelgrass presence) vary across the Puget Sound estuary, and that species’ interactions with aquaculture might also vary^4–6^. The habitat types for this study were defined based on the cultured species, grow-out method, and substrate type: Pacific oysters grown on the sediment (hereafter referred to as to as ‘oysters on bottom’), Pacific oysters in suspended flipbags (hereafter referred to as ‘oysters in flipbags’), Manila clams grown in the sediment under anti-predation nets (hereafter referred to as ‘clams’), and ‘other’, which was used to group sites that were a mix of the other standardized habitat sites and geoduck sites. Individually, the habitats in the ‘other’ category represented too small a sample size to warrant their own habitat category. These habitats may have been unique to a certain site or reflected short-term or interim habitat types. Examples of these standardized habitat types are indicated in the side panel in Fig. 1. In addition to the five aquaculture mesohabitat types, two types of reference mesohabitats (where no aquaculture was present) were included at each farm, where available. Reference sites were categorized as eelgrass (*Z. marina* or *Z. japonica*) or sediment (a range of mudflats to more gravelly beaches, sometimes with low-density eelgrass, *Z. marina* or *Z. japonica*, present), depending on the prominent mesohabitat characteristics. In regions where eelgrass was present (North Sound and Hood Canal), oysters in flipbags were often located at depths in which eelgrass would naturally occur, while clams and oysters on bottom were often in shallower, more naturally sediment-dominated, or gravelly habitats. These sites were 30 to 60 m away from existing aquaculture and represented the range of natural habitats in the area. This distance was selected to maximize the similarity in environmental conditions and depth but minimize potential effects from aquaculture sites. Cameras were mounted on two PVC poles, which were placed 125 cm from the base of the camera pole and 1 m apart, so that the field of view was 1 m^2^ (marked by two more PVC poles). The distance of 125 cm compensated for the extra 25 cm of off-camera area created because of the angle of the camera.

Images extracted from these videos were labeled to indicate the presence or absence of fishes or crabs (denoted as animals) and the coordinates of bounding boxes are available for images that contained animals. Our data set has been archived on Mendeley Data^7^ (under the title NOAA Puget Sound Nearshore Fish 2017–2018) and can also be accessed as one of the data sets in the Labeled Information Library of Alexandria: Biology and Conservation (LILA BC).

**Fig. 2:**
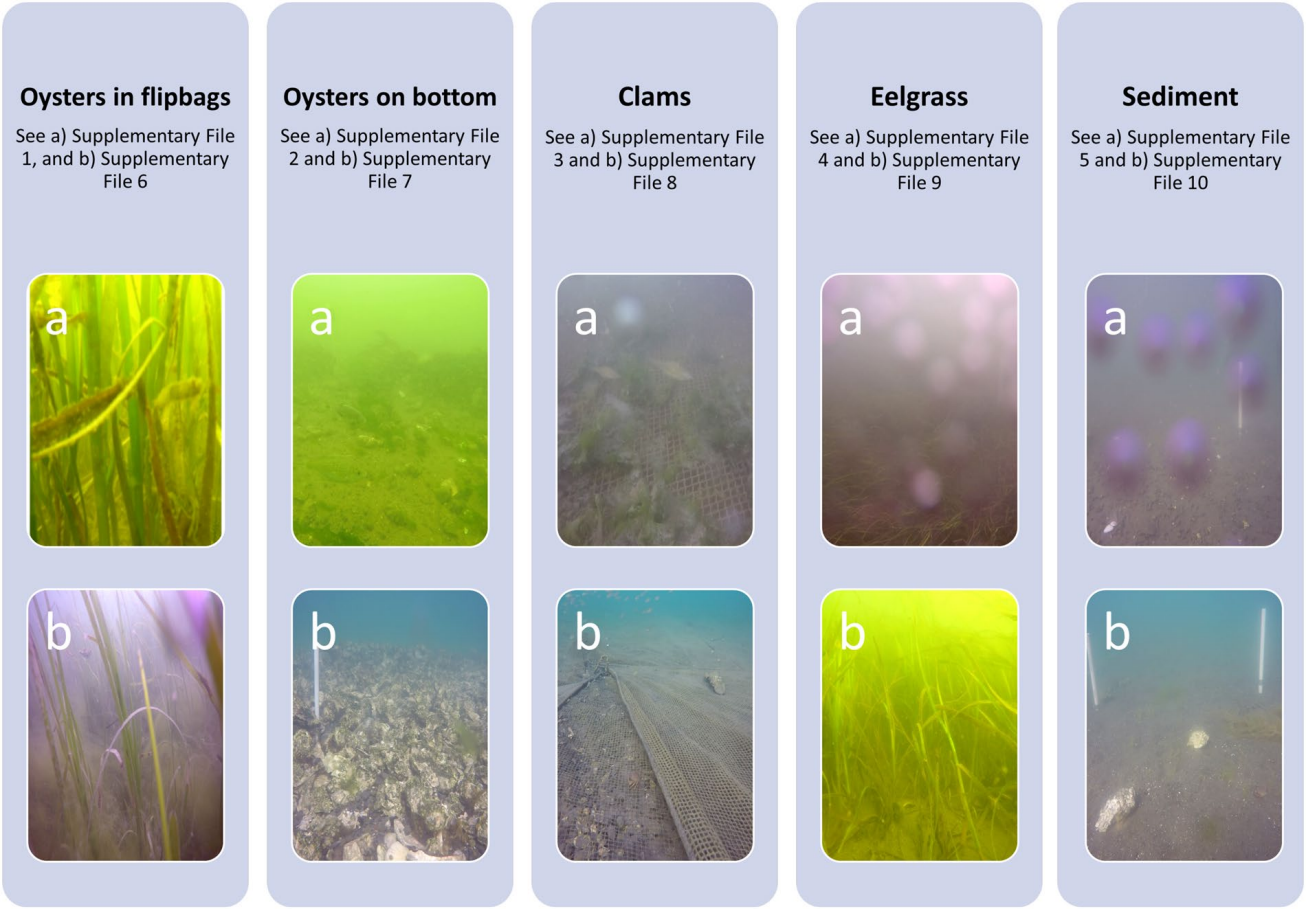
Examples a (from Supplementary files 1–5) and b (from Supplementary files 6–10) of the range of image quality for each habitat type. Note that the Oysters in flipbags images (both a and b) do not show the flipbags themselves since they were above the field of view. Examples of cases where bubbles obstructed the camera view are also shown (Eelgrass (a) and Sediment (a)).

Other researchers have made labeled fish data sets in underwater environments publicly available, and a curated list is available from LILA BC, which includes brackish-water data sets such as the work by Cutter *et al*.^8^, (as well as other conservation data sets); three are highlighted in Table 1. Our data set adds to the available data corpus by contributing additional underwater images, without artificial lighting, of fish and crab species that are endemic to the Pacific Northwest region of the United States. They also provide examples of eelgrass-dominated and muddy environments, geoduck cultivation locations, clam cultivation locations, and oyster cultivation locations (on bottom and in flipbags). Water clarity varied because of high algae concentration or the presence of bubbles that obscured the camera lens as shown in Fig. 2. For particular ecological studies, such as studies of animal behavior, it is desirable to minimize changes to the natural environment when collecting data, which discourages the use of artificial light sources. Data sets that feature brackish water are rare, and naturally lit examples showcasing field data collection challenges are even more rare and will allow for the training of models that are able to support such studies. The inclusion of these difficult field conditions may make such algorithms more robust.

**Table 1:** Examples of additional data sets with images of varying visibility.

Citation
Pedersen, M. *et al*. “Detection of marine animals in a new underwater dataset with varying visibility”. Proceedings of the IEEE/CVF Conference on Computer Vision and Pattern Recognition Workshops, 2019.
Islam, M.J. *et al*. “Semantic segmentation of underwater imagery: Dataset and benchmark”. 2020 IEEE/RSJ International Conference on Intelligent Robots and Systems (IROS). IEEE, 2020.
Pedersen, M. *et al*. “BrackishMOT: The Brackish Multi-Object Tracking Dataset”. Image Analysis: 23rd Scandinavian Conference, SCIA 2023, Sirkka, Finland, April 18–21, 2023, Proceedings, Part I. Cham: Springer Nature Switzerland, 2023.

Additional examples can be accessed from LILA BC.

## Methods

**Fig. 3:**
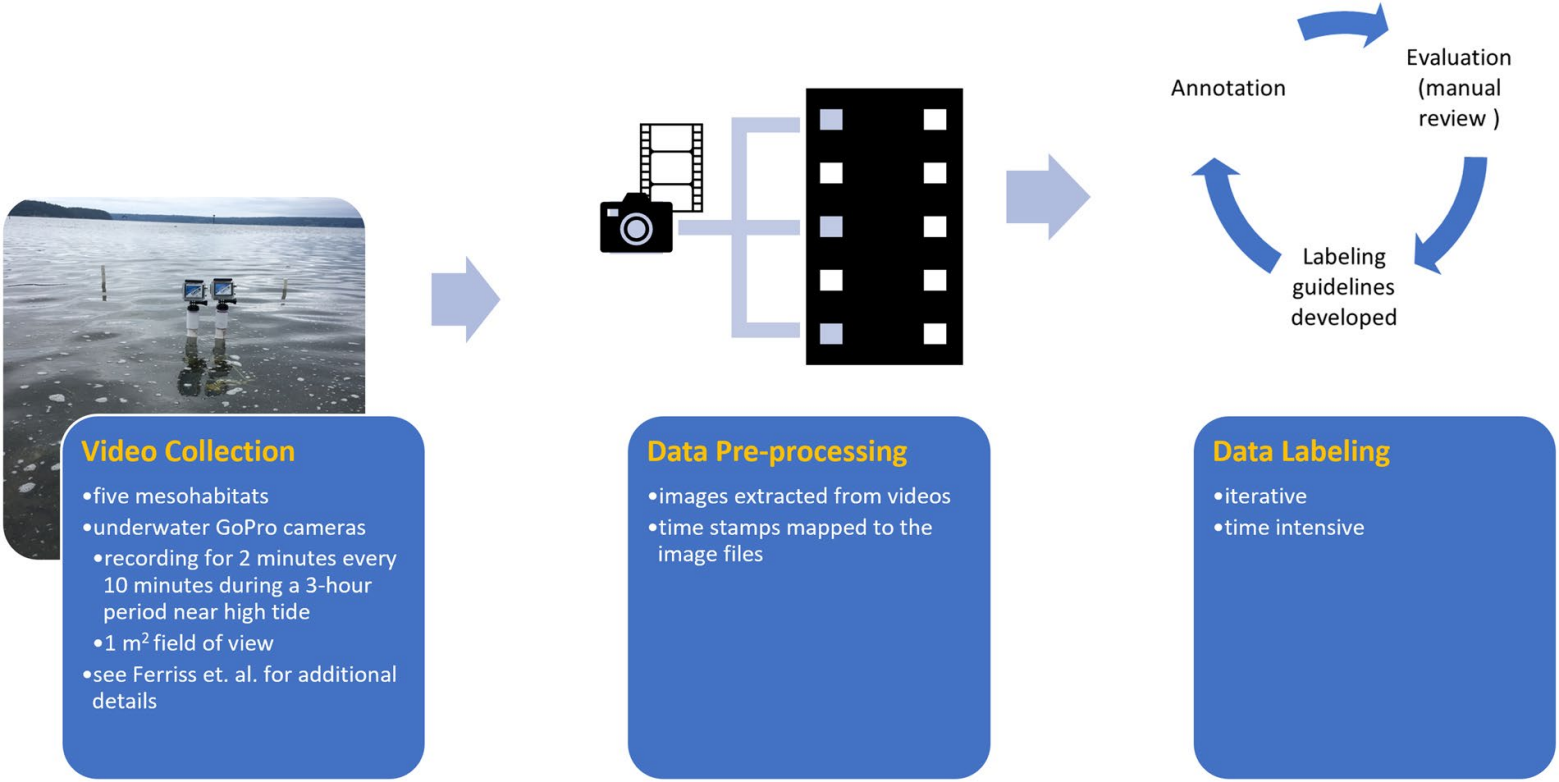
Phases of production of the labeled data set.

The production of this labeled data set was a multi-stage process, which is illustrated in Fig. 3. Each phase is summarized, and further details for each phase are supplied below.

### Video collection

**Fig. 4:**
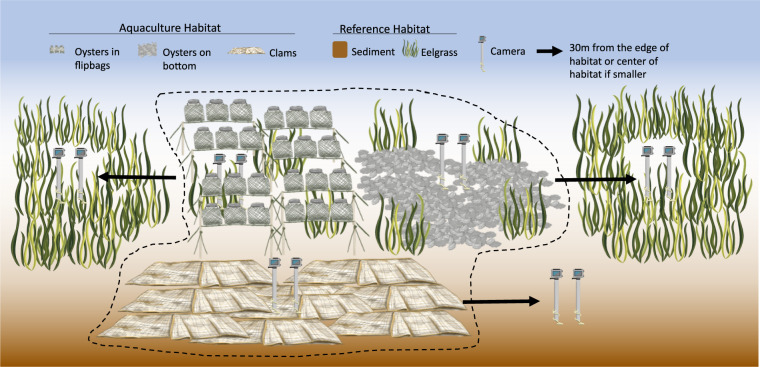
Camera locations in aquaculture (oysters in flipbags, oysters on bottom, clams) and reference (sediment, eelgrass) mesohabitats for a given aquaculture farm (dashed line) during a single sampling event. Two cameras were set in each mesohabitat for every sampling event with a field of view of 1 m^2^. Reference sites were 30 m away from the edge of aquaculture habitat but at similar depth and other environmental conditions. On certain farms, the same site served as a reference for multiple aquaculture sites if available reference areas were limited and the habitats were similar. Adapted from a figure previously published in Ferriss *et al*.^2^.

In collaboration with shellfish growers, underwater GoPro cameras (models GoPro Hero 3+ and Hero 4) were used to document crabs (e.g. Dungeness crabs), and nearshore fishes (including outmigrating salmonids) in both shellfish aquaculture and uncultivated nearshore habitats from 6 farm locations (in North Sound and South Sound) twice per summer from June to August 2017, and at 9 farm locations from June to August 2018. In Fig. 1, full circles indicate that a given site was sampled in both years, and half-filled circles indicate that the given site was only sampled in 2018. These locations were distributed throughout North Sound, South Sound, and Hood Canal. Five mesohabitat types (visually distinct habitats) in Puget Sound are present in the data set, which are shown in the side panel of Fig. 1. Three aquaculture sites and two reference sites are included in the data set; however, there were no eelgrass sites in South Sound. Figure 4 illustrates the positioning of the cameras at a given location (the 9 locations sampled in the study area shown in the main panel of Fig. 1). Two cameras were placed facing the incoming tide since this was the direction of best visibility.

Cameras were generally deployed for 2–3 days (to ensure a minimum of 48 hours of data). They would typically be deployed at low tide on the first day (Day 1). They would be set to turn on to record at high tide that day, and then again at high tide on Day 2. The cameras recorded for 2 minutes every 10 minutes during a 3-hour period near high tide. They were then collected on the third day at low tide for necessary maintenance, such as changing batteries and sim cards. The cameras would then be redeployed in the following month at the sites.

More than 1200 hours of video were collected from the 9 shellfish aquaculture locations around Puget Sound, WA. Videos from select sites in 2017 had a predominantly green background because of high algae concentration in the water; therefore, in 2018, the GoPro cameras were outfitted with magenta filter caps (PolarPro) to improve visibility in the videos. Occasionally field video was obscured by weather or environmental conditions that caused bubbles to collect between the filter and the camera lens.

### Data pre-processing

The videos that were collected from each sampling date, farm, and habitat type (eelgrass, sediment, oysters on bottom, oysters in flipbags, and clams) resulted in a data set of approximately 200,000 videos. Images were extracted from these videos, and time stamps were mapped to the image files prior to annotation of animals. Approximately 60% of the images in the data set were from videos in 2018 where magenta filter caps were used. The approximate percentage of images by habitat type is documented in Table 2, and the approximate percentage of images by animal type is given in Table 3.

**Table 2:** Approximate distribution of labeled image data set by habitat type.

Habitat Type	Percentage of images (% in data set)	Number of images
sediment	16.4	12,800
eelgrass	13.3	10,600
oysters in flipbags	28.1	21,800
oysters on bottom	17.9	13,900
clams	14.8	11,500
other	9.4	7,200

Percentages are based on the 30,384 images tagged as containing animals and have been rounded to one decimal place with a rounding error of approximately 0.1%; the corresponding number of images has been rounded to the nearest hundred.

**Table 3:** Approximate distribution of labeled image data set by animal type; percentages are based on the 30,384 images tagged as containing animals.

Animal Type	Percentage of images (% in data set)	Number of images
crab	16.1	4,900
fish	74.6	22,700
both	8.4	2,600

Percentages have been rounded to one decimal place and the number of images has been rounded to the nearest 100. A small percentage ( < 1%) was neither fish nor crab.

### Data labeling

Data labeling (or data annotation) describes the process of adding labels (or annotations) to data to provide context for later use in training machine learning models. A high-quality labeled data set is the result of an iterative and exhaustive process, and the goal for this data set was an accuracy of greater than 95% on both an image and data set level.

**Fig. 5:**
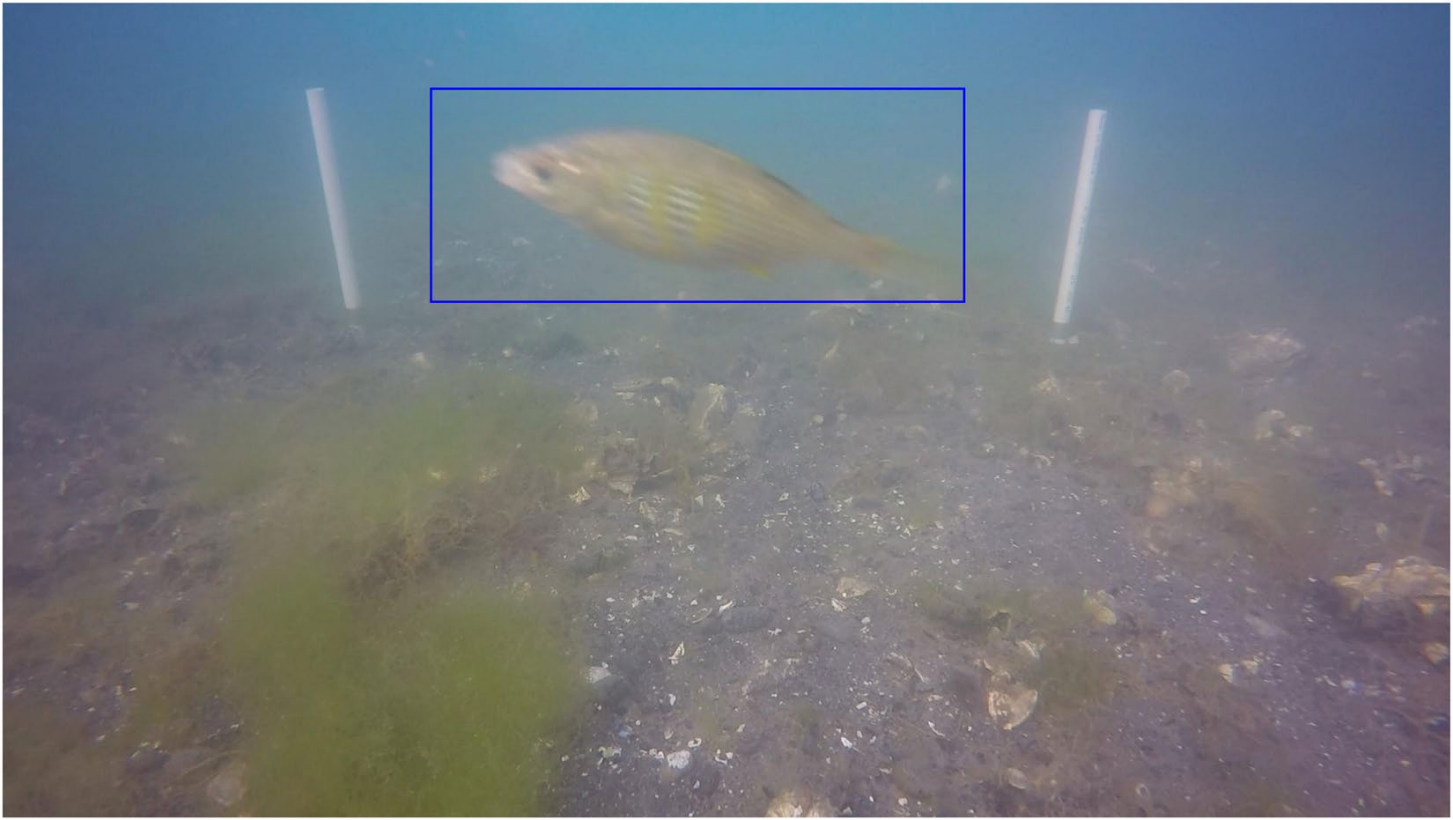
Example of bounding box annotation. The white poles in the background mark the 1 m^2^ field of view.

More than a dozen persons formed the labeling team for this project. The annotators used labeling software (a proprietary tool created by iMerit), which featured the ability to perform task-based labeling and had the core features that allowed the annotators to: 1) group frame-level sub-tasks within a larger task, so that a single person would be responsible for reviewing a video in its entirety; 2) easily navigate forward and backward between frames in order to more easily detect changes between frames, or movement related to the presence of difficult to detect fish; 3) label objects of interest with attributes and bounding boxes (as shown in Fig. 5); and 4) produce a standardized JSON output mapping of frames to annotation metadata. The frames of the videos were annotated at a predetermined sampling rate during pre-processing. The labeling exercise was initiated with a series of small calibration batches to ensure consistency in the alignment of tasks and the quality standards required from the labeling team. Two individuals annotated the frames for each video in its entirety; one person performed a first-pass annotation and the second performed a quality-check pass and edited any discrepancies. Images from the oysters on bottom sites were particularly challenging to annotate. These sites attract many small crabs that are well camouflaged. Users should be aware that annotations for these habitats likely underestimate the presence of crabs.

After this annotation process was completed, an additional label was created to indicate whether a magenta filter cap was used in the data collection. This label was inferred by matching portions of the file names of the images to the video collection location notes. The addition of this label to the final data set was manually audited for accuracy. Inconsistencies or errors discovered in the labeled data were used to further refine the labeling process.

## Data Records

The open-source data set is available for download from Mendeley Data^7^ (under the title NOAA Puget Sound Nearshore Fish 2017–2018) and can also be accessed from LILA BC.

This approximately 7 GB data set contains 77,739 images sampled from the collected videos; 67,990 objects (primarily fishes and crabs) have been annotated in 30,384 images. The remainder of the images (47,355 images) have been annotated as “empty”. All images (with and without animals) are at a resolution of 1920 × 1080 pixels.

Annotations are provided in a widely used format for computer vision data sets, (the COCO Camera Traps JSON format). The provided JSON file contains the following key-value pairs:

- info: This key corresponds to a dictionary that contains metadata about the data set.
  - version: The version of the data set.
  - description: A brief description of the data set.
  - year: The year the data set was uploaded.
  - contributor: The entity that contributed to the data set (‘NOAA Fisheries’).
- annotations: This key corresponds to a list of dictionaries.
  - id: A unique identifier for the annotation.
  - image_id: The identifier for the image associated with this annotation.
  - category_id: The identifier for the category associated with this annotation.
  - sequence_level_annotation: A Boolean value that indicates whether the annotation is at the sequence level.
- images: This key is linked with a list of dictionaries. Each dictionary represents a single image:
  - width: The width of the image in pixels.
  - height: The height of the image in pixels.
  - file_name: The name of the image file.
  - location: The location associated with the image.
  - id: A unique identifier for the image.
  - filter: A Boolean value that indicates whether a magenta filter cap was used in the collection of the video associated with the image.

- categories: This key also corresponds to a list of dictionaries. Each dictionary represents a single category:
  - id: A unique identifier for the category (0 or 1).
  - name: The name of the category.
  Note that locations have been replaced with random IDs to preserve the confidentiality of the local farms.
  For example, consider the example below showing typical entries for an empty image:
- ‘annotations’:
  - ‘id’: ‘bc8df6be-1207-11ed-a46d-5cf3706028c2’,
  - ‘image_id’: ‘SD11_227_6_26_2017_1_73.73.jpg’,
  - ‘category_id’: 0,
  - ‘sequence_level_annotation’: False

- ‘images’:
  - ‘width’: 1920,
  - ‘height’: 1080,
  - ‘file_name’: ‘SD11_227_6_26_2017_1_73.73.jpg’,
  - ‘location’: ‘loc_bc8df6bd-1207-11ed-8899- 5cf3706028c2’,
  - ‘id’: ‘SD11_227_6_26_2017_1_73.73.jpg’,
  - ‘filter’: False
- ‘categories’:
  - ‘id’: 0,
  - ‘name’: ‘empty’

## Technical Validation

Labeled data were evaluated against set criteria to ensure that all labeled data were annotated in accordance with the same set of rules, such as the object classes requested, the tightness of the bounding boxes, and the treatment of ambiguous cases. Annotators used consistent definitions for each object (fishes and crabs) and labels were cross-checked (as discussed in the Methods section); ambiguous cases were labeled by consensus. After annotation of fishes and crabs, the labeled data set was verified manually. Inconsistencies or errors discovered in the labeled data were used to further refine the labeling process. The source data that was used was sufficiently broad in scope and quantity to be representative of real-world conditions.

It is important to note that this video data set is restricted by a limited field of view^9–11^. The downward angle of the pair-placed cameras and timing of the recordings may have missed fishes coming in on the leading edge of the tide (i.e., in the first waters of the tide) since recordings were made at slack tide (i.e., when currents were weakest) once the tide was already in and before it receded.

## Usage Notes

Users should note that some species were well represented in the data set, but many were not. Although this data set does not include a species label, there are numerous species that have large sample sizes of observations across multiple regions in Puget Sound. Users can expect to see examples of some of the higher sample size species: surf perch (*Embiotocidae*), various crabs (*Hemigrapsus* and juvenile *Metacarcinus*), sculpins (*Cottidae*), and flatfish^2^).

These kinds of data sets contribute to broader applications related to habitat changes due to climate change or human impacts^2,12,13^. This data set (and similar) also has the potential to address questions related to estuarine/ nearshore marine ecosystems at the system level. They can be applied to more in-depth regional shellfish aquaculture questions related to ecosystem services and interactions of shellfish aquaculture. Ferriss *et al*.^2^ and Shinn *et al*.^12^ used free and open-source video editing software in their work to analyze subsets of video data (BORIS Behavioral Observation Research Interactive Software). Computer vision models would be helpful in expanding video analysis capability through automation in these cases, and a variety of water conditions (brackish to clear) would make such models more robust.

It is likely that researchers who seek to use this data set to train models will be best served by augmentation with other labeled data–particularly for underrepresented species. Additionally, ensuring that the training data set is balanced with respect to the image background/habitat type may be helpful. Despite these caveats, this data set’s representation of naturally-lit habitats should be useful in improving machine learning model performance under brackish water conditions.

### Supplementary information

Supplementary Files

## Data Availability

This is an image data set and annotations have been provided in a widely used format for computer vision data sets–the COCO Camera Traps JSON format. Since the annotations have already been included in a standardized format, no additional code has been supplied.
