# Supplementary material for: A labeled data set of underwater images of fish and crab species from five mesohabitats in Puget Sound WA USA
Source: Sci Data. 2023 Nov 13;10:799. doi: 10.1038/s41597-023-02557-6 (PMC10643608; doi:10.1038/s41597-023-02557-6)
Supplement: Supplementary file 1 — Supplementary Files [file 41597_2023_2557_MOESM1_ESM.zip › Supplementary Files/Readme.rtf]

All Supplementary files have been referenced in Figure 2 as the source of the a and b clips for each habitat type.
